# An Adrenal Mass and Increased Catecholamines: Monoamine Oxidase or Pheochromocytoma Effect?

**DOI:** 10.14740/jocmr2042w

**Published:** 2014-12-29

**Authors:** Marianne R. F. Bosscher, Iris M. Wentholt, Mariette T. Ackermans, Els J. M. Nieveen van Dijkum

**Affiliations:** a Department of Surgery, Academic Medical Center, University of Amsterdam, Amsterdam, The Netherlands; b Department of Internal Medicine and Endocrinology, Amphia Medical Hospital, Breda, The Netherlands; c Department of Clinical Chemistry, Academic Medical Center, University of Amsterdam, Amsterdam, The Netherlands

**Keywords:** Pheochromocytoma, MAO inhibitor, Pseudopheochromocytoma, Hormonal screening

## Abstract

Hormonal evaluation in patients with an adrenal incidentaloma can be difficult in patients with comorbidities or in patients using interfering drugs. We present a case of a 54-year-old man who was evaluated for an adrenal mass. The medical history reported treatment with a monoamine oxidase (MAO) inhibitor for recurrent psychoses. Hormonal screening showed elevated levels of normetanephrine and metanephrine in plasma and urine, suggesting a diagnosis of pheochromocytoma (PHEO), and an adrenalectomy was performed. Histologic examination showed that the tumor had an origin of the adrenal cortex. MAO inhibitors are also known to cause elevated levels of catecholamines. In this case, a PHEO seemed more likely the cause due to repeatedly elevated levels of metanephrines and normal levels of catecholamines. Since the tumor had an origin of the adrenal cortex, the use of MAO inhibitors was the most likely explanation for the elevated levels of metanephrines. This case illustrated the difficulties in diagnosing PHEO, especially in patients with comorbidities and interfering drugs.

## Introduction

Since the introduction of imaging techniques in medical practice, abnormalities of unknown clinical significance are found more often. One of these abnormalities is the accidently discovered adrenal tumor or adrenal incidentaloma [1]. In order to differentiate the adrenal mass detected on abdominal imaging studies, patients should always be evaluated with hormonal activity measurements [2]. This hormonal evaluation can be difficult in patients with comorbidities or in patients using interfering drugs. This case report illustrates the difficulties of interpreting adrenal hormone function tests in a patient using monoamine oxidase (MAO) inhibitors.

## Case Report

A 54-year-old man presented with pain in the right abdomen and hematuria for 10 days. The medical history reported alcohol abuse, depression and episodes of acute psychosis. Because of a resistance to conventional medication, such as selective serotonin reuptake inhibitors (SSRIs), he was treated with Tranylcypromine Sulphate (Parnate^®^), an MAO inhibitor.

The hematuria and abdominal pain resolved spontaneously within 14 days. Further questioning of the patient showed that he had complaints of excessive perspiration, nervousness and tachycardia, although he referred these complaints to anxiety during psychotic episodes. There were no typical paroxysms including forceful heart beat, hypertension, pallor, tremor or headache. During the workup ultrasonography revealed a mass in the left upper abdomen, and an additional CT scan showed an oval lesion in the left adrenal gland with a maximum diameter of 3.2 cm and 44 Houndsfield units (HU, reference value < 10 HU for benign lesions). There were no other abnormalities in the abdomen and no signs of distant metastases.

Hormonal screening of the adrenals was performed multiple times. The aldosterone, cortisol and testosterone levels were within reference values as well as plasma adrenaline and noradrenaline levels. However repeatedly, there was an elevated level of plasma normetanephrine (1.89 nmol/L, reference value < 0.60 nmol/L ) and elevated levels of fractioned metanephrine and normetanephrine levels in the urine (1.67 μmol/24 h and 16.86 μmol/24 h respectively, reference values < 1.52 μmol/24 h and < 3.26 μmol/24 h respectively).

To determine whether the elevated catecholamine level in the urine was caused by the MAO inhibitor or an adrenal pheochromocytoma (PHEO), the medication should be temporarily withdrawn. However, the patient was too anxious for a relapse of his psychosis that he insisted on sustainment of the pharmacological treatment. Because of a great lack of uncertainty about the origin of the elevated metanephrine levels, and the risk of a malignant tumor in the abdomen given the high HU on imaging, the choice for left adrenalectomy was made. The patient was admitted to the hospital 5 days prior to surgery for preparation with alpha adrenergic antagonists to accomplish an optimal blood pressure and heart rate. Intraoperatively, the patient remained stable without any hypertensive crises.

Histologic examination of the surgical extract revealed a benign adenoma originating from the adrenal cortex, and not the medulla. The postoperative recovery was without complications and was discharged 1 day postoperatively. A few weeks later during the postoperative visit at the outpatient clinic, the patient mentioned an increased energy level and less anxiety feelings compared to before the adrenalectomy.

## Discussion

A PHEO is a very rare tumor of the adrenal medulla [3]. The incidence is between two and eight cases per million per year [4]. The tumor secretes catecholamines that cause sustained or intermittent hypertension with associated symptoms of headache, flushing, sweating, anxiety, dizziness and palpitations [3, 5, 6]. Between 70% and 80% are found sporadically and about 10% of adrenal PHEOs are found to be malignant [3, 5].

In the adrenal medulla, catecholamines (adrenaline, noradrenaline, and dopamine) are secreted and converted to metadrenalines (metanephrines, normetanephrine, and 3-methoxytyramine) [5]. In this way, the adrenal medulla is responsible for about 90% of the plasma metanephrine, and 35% of the plasma normetanephrine [7]. On the other hand, only about 10% of the circulating adrenaline is secreted by the adrenal medulla, while almost 90% of the plasma noradrenaline is secreted by sympathetic nerves [7]. The secretion of noradrenaline by sympathetic nerves is influenced by stress and many drugs [5]. In a PHEO (originating from the medulla), there is an increased metabolism of catecholamines to metadrenalines, and therefore patients can have a high level of free metadrenalines in plasma and normal levels of catecholamines [4]. For this reason, testing plasma free metadrenalines is more sensitive and specific than testing catecholamines in the diagnostic process of a PHEO [8].

The only definitive treatment for PHEO is surgical adrenalectomy [3]. Surgical resection of the tumor requires intensive intraoperative hemodynamic management because of the risk of hypertensive crisis and death when causing the tumor to leak a large amount of catecholamines or the risk of intraoperative bleeding after removal of the tumor [3, 9].

MAO inhibitors reduce monoamine degradation of neurotransmitters such as serotonin and noradrenaline in the synaptic cleft [10]. The use of MAO inhibitors is associated with severe side effects such as raised plasma levels of noradrenaline causing acute hypertensive attacks, and therefore the use of this type of medicine has been eliminated from common practice [10, 11]. Patients taking MAO inhibitors require a tyramine restrictive diet, another sympathicomimeticum (found in fermented foods), of which the metabolism will be inhibited by MAO inhibitors.

A pseudopheochromocytoma has been described as a rare syndrome with paroxysmal hypertension and symptoms of catecholamine excess [12]. However, in most cases there is no anatomical or biochemical explanation for these symptoms, although increased dopamine as well as adrenal epinephrine release and increased circulatory response to catecholamines have been reported particularly during paroxysms [12, 13]. A pseudochromocytoma has been described after taking MAO inhibitors [14, 15]. In these cases there are extremely high plasma catecholamines, and they disappear after eliminating the drugs.

MAO inhibitors are contraindicated in patients with or on suspicion of a PHEO. When there would be a hypertensive crisis due to excess release of catecholamines by the PHEO, the clearance of circulating catecholamines would be reduced by the MAO inhibitor [9, 16]. When there are severe hypertensive episodes in patients taking MAO inhibitors regardless of a dietary restriction of tyramine, a PHEO should be suspected [16].

In this case the patient repeatedly had elevated levels of plasma free metadrenalines whereas there were normal levels of catecholamines. Second, on imaging the adrenal mass had 44 HU. For these reasons, the suspicion of a PHEO was high. Preferably, the patient’s medication was stopped (temporarily), to evaluate the symptoms and the patient’s plasma levels of catecholamines for differentiation between a PHEO and side effects of the MAO inhibitor. This would have saved the preoperative precautions taken for PHEO-related surgery, and possibly even an unnecessary surgical intervention. Unfortunately, in the communication with the patient this was not an option, since he refused to quit his antidepressant medication. The patient’s wish was accepted and there was no other option than treating the adrenal mass as being a PHEO.

### Conclusion

This case illustrates the difficulties in diagnosing PHEO, especially in patients with comorbidities and interfering drugs. The findings of elevated levels of metadrenalines in blood and urine in any patient would lead to a diagnosis of PHEO [4]. However, MAO inhibitors are also known to cause elevated levels of catecholamines and (hypothetically) increased metabolism to metadrenalines [10]. Since the patient repeatedly showed elevated metanephrine and normetanephrine levels, a PHEO was most likely to be the cause of the elevated levels of these metabolites. This is why the decision for a surgical intervention, an adrenalectomy, was made. Eventually, the tumor had an origin of the adrenal cortex. Retrospectively, we can conclude that the use of MAO inhibitors was the most likely explanation for the elevated levels of metadrenalines.
